# A neurovascularized bone regeneration strategy for mandibular and alveolar bone defects based on elastin-like biomaterials

**DOI:** 10.1093/rb/rbag036

**Published:** 2026-03-05

**Authors:** Nadia Mahmoudi, Romane Lesieur, Sylvie Rey, Bruno Paiva Dos Santos, Sylvain Catros, Bertrand Garbay, Joëlle Amédée Vilamitjana, Micaela Roque

**Affiliations:** Tissue Bioengineering Laboratory (BioTis), Inserm U1026, University of Bordeaux, Bordeaux 33000, France; Tissue Bioengineering Laboratory (BioTis), Inserm U1026, University of Bordeaux, Bordeaux 33000, France; Tissue Bioengineering Laboratory (BioTis), Inserm U1026, University of Bordeaux, Bordeaux 33000, France; Tissue Bioengineering Laboratory (BioTis), Inserm U1026, University of Bordeaux, Bordeaux 33000, France; URP2496-BRIO Pathologies Imagerie et Biothérapies Orofaciales, Université Paris Cité, Montrouge F-92120, France; Tissue Bioengineering Laboratory (BioTis), Inserm U1026, University of Bordeaux, Bordeaux 33000, France; Dentistry and Oral Health Department, CHU Bordeaux, Bordeaux 33076, France; CNRS, Bordeaux INP, CBMN, UMR 5248, Université de Bordeaux, Pessac F-33600, France; Tissue Bioengineering Laboratory (BioTis), Inserm U1026, University of Bordeaux, Bordeaux 33000, France; Tissue Bioengineering Laboratory (BioTis), Inserm U1026, University of Bordeaux, Bordeaux 33000, France

**Keywords:** biomaterial, craniofacial defects, bone regeneration, elastin-like polypeptides

## Abstract

The regeneration of craniofacial bone defects requires biomaterials that provide more than mechanical support, enabling coordinated osteogenesis, angiogenesis, and innervation. To address this challenge, we designed a composite scaffold based on elastin-like polypeptides (ELPs) functionalized with bioactive peptides to recruit endothelial and sensory neuron cells, allow degradation, and ensure hydroxyapatite distribution for enhanced mineralization. The scaffold was evaluated in two critical-size defect models, the rat mandible and minipig alveolar bone, promoting progressive bone formation, robust vascularization, neural infiltration, and controlled immune response. In rats, the ELP scaffold achieved 36% bone fill at 4 weeks, compared with 24% for the control material Collapat^®^ and 16% in untreated defects, accompanied by dense vascular and neural networks. Immune cell infiltration was also significantly reduced relative to Collapat^®^, indicating improved immunotolerance. In minipigs, ELP-treated defects showed 36% new bone formation after 8 weeks, characterized by well-organized lamellar bone, integrated osteocytes, and mature neurovascular networks. Compared with commercial matrices, the ELP scaffold consistently demonstrated superior outcomes in bone formation, tissue integration, and functional innervation. Overall, this biomaterial promotes synchronized neurovascularized bone regeneration while maintaining excellent biocompatibility, remodeling capacity, and regenerative efficacy, making it a promising candidate for mandibular and alveolar bone repair.

## Graphical Abstract

**Graphical Abstract:**
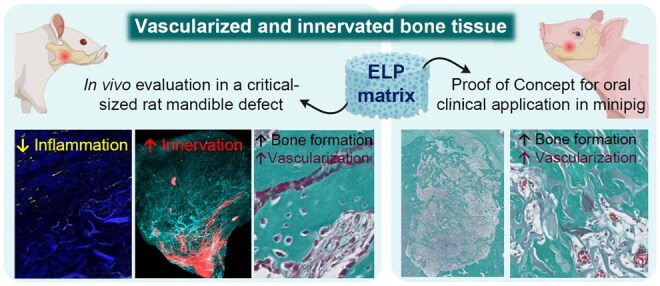

## Introduction

Craniofacial and dental bone defects present a significant challenge in regenerative medicine, as these regions are essential for functions such as chewing, speaking, and maintaining aesthetic facial structure [1]. The alveolar bone, in particular, plays a critical role in dental implant stability and oral function, making its regeneration key to achieving successful outcomes in dentistry. In cases of severe trauma, periodontal disease, or surgical resection, the resulting defects often exceed the bone’s intrinsic healing capacity, requiring clinical intervention to restore both structural and functional integrity [2].

Craniofacial bone regeneration in these areas poses unique challenges due to the complex anatomical structures, thin bone layers, and the need for precise functional restoration [3]. Critical-sized defects, defined as those incapables of spontaneous healing, often require advanced surgical and biomaterial-based interventions to achieve satisfactory outcomes [4, 5]. There is no universally accepted definition of a critical-sized bone defect, and reported values vary considerably depending on the anatomical location of the defect and the condition of the surrounding soft tissues. In clinical orthopedics, such defects are generally defined as bone defects that do not heal spontaneously despite adequate surgical stabilization and therefore require bone grafting, typically corresponding to a defect length of approximately 2 cm in humans or involving more than 50% of the bone circumference [6].

Traditional approaches, including autografts, allografts, and xenografts, remain the standard for addressing craniofacial bone defects [7]. However, these methods are associated with significant limitations such as donor site morbidity, limited availability of autologous grafts, and immunogenic risks with allografts [8]. Furthermore, these strategies often trigger a significant inflammatory phase, which can delay healing, compromise graft integration, and exacerbate the risk of graft rejection [9]. Tissue engineering strategies have emerged as promising alternatives, leveraging scaffolds, growth factors, and cell therapies to enhance bone regeneration [10]. Despite significant advancements, current materials often fall short in replicating the complex bone microenvironment, resulting in suboptimal vascularization and innervation, both of which are crucial for functional integration and long-term success [11]. These limitations highlight the pressing need for innovative biomaterials tailored to craniofacial and dental applications, capable of addressing the multifaceted requirements of vascularized and innervated bone regeneration [12, 13].

Bone regeneration proceeds through a tightly regulated sequence of events, starting with inflammation, followed by tissue repair and structural remodeling. During the initial inflammatory phase, dilation of blood vessels in the periosteum and adjacent tissues enhances vascular permeability and immune cell recruitment, such as macrophages and neutrophils [14, 15]. These cells release cytokines and growth factors, including vascular endothelial growth factor (VEGF), that trigger angiogenesis at the injury site [16, 17].

This newly established vascular network ensures the supply of nutrients, oxygen, and signaling molecules essential for the recruitment and osteogenic differentiation of mesenchymal stem cells (MSCs) [18]. Activated by VEGF, endothelial cells (ECs) proliferate and migrate into the defect, forming new capillaries that infiltrate the hematoma [19]. It also provides a structural framework that guides osteoprogenitor migration and supports matrix deposition.

In parallel, the nervous system contributes to the modulation of bone cell activity through the release of neuropeptides and neurotransmitters, which regulate cellular crosstalk within the bone microenvironment. Innervation, often closely associated with the vasculature [20, 21], is also essential as it impacts local bone remodeling, the quality of newly formed bone [22], and therefore clinical outcomes. Nerve fibers release neuropeptides such as calcitonin gene-related peptide (CGRP) and substance P, which modulate osteoblast and osteoclast activity [23]. CGRP, for instance, promotes osteoblast differentiation and matrix mineralization [24], whereas substance P enhances the recruitment of ECs and stimulates local angiogenesis [25]. Studies in preclinical models have demonstrated that the absence of sensory innervation delays the transition from the cartilage callus to the bony callus, a crucial step in endochondral ossification [26, 27]. This is also supported by clinical data showing that patients with insufficient peripheral innervation often exhibit impaired bone repair and an increased risk of recurrent fractures [28]. However, despite these data, most current commercial scaffolds and regenerative therapies remain primarily focused on osteoblast activity and mineralization, often overlooking the equally essential processes of vascularization and innervation.

These limitations are particularly relevant in craniofacial bone repair, where the interactions between the periosteum, mucosa, and underlying bone require a coordinated neurovascular response for successful regeneration. Insufficient vascularization leads to necrotic regions within the graft, while inadequate innervation reduces the mechanical and functional integration of the newly formed bone [29]. To overcome this, biomaterials must actively promote neurovascular coupling, ensuring the synchronized formation of vascular and neuronal networks within the newly formed bone.

Recently, Zhang *et al*. have published the development of a polyhedron-like biomaterials for innervated and vascularized bone regeneration and tested *in vivo* their efficacy in a rabbit femoral defect models [30]. However, proving the effectiveness of such biomaterials requires robust and relevant preclinical testing to validate their capacity to support these processes *in vivo*. Among preclinical experimental models, the rat mandibular defect model is often selected for its ability to provide a reproducible platform for studying early stage vascular and neuronal ingrowth within a compact anatomical site. This model is subject to mechanical stresses and can also provide a sufficient number of samples required to obtain significant differences compared to the positive controls. In addition, for translational relevance, the alveolar defect model in a large animal such as the minipig was chosen due to the anatomical and physiological similarities of porcine craniofacial bones to those of humans [31]. The defect size and bone properties in minipigs closely mimic patient clinical conditions, offering an excellent surrogate for evaluating the scalability and feasibility of biomaterials in similarly sized defects before clinical trials [32, 33]. This model also reflects the challenges of craniofacial bone regeneration, including the intricate interplay of the periosteum, mucosa, and alveolar bone.

Building on the previously discussed challenges in bone regeneration and the critical role of vascularization and innervation, it becomes evident that addressing these limitations requires innovative biomaterials capable of supporting these essential physiological events. In this context, the elastin-like polypeptide (ELP)-based composite biomaterial developed in our previous study [34], represent a significant step forward for tissue engineering. This fully composite matrix is functionalized with biomimetic peptides IKVAV and YIGSR to enhance cell adhesion, along with the SNA15 peptide to optimize hydroxyapatite (HA) particle retention and distribution within the polymer. This matrix has demonstrated porosity and interconnectivity, suitable for the culture of MSCs, ECs, and sensory neurons (SNs) [34]. *In vitro* evaluations revealed that the IKVAV/YIGSR peptides played a crucial role in promoting neurite outgrowth in SNs. Finally, *in vivo* studies on ectopic and condylar rodent models showed that the matrix effectively stimulated osteoid tissue formation, bone mineralization, and the development of vascular and neural networks while eliciting minimal inflammatory responses [34].

Based on these findings, we now evaluate this biomaterial in two clinically relevant models, providing translational insight into its regenerative potential. The rat model assesses early neurovascular ingrowth and biocompatibility/inflammatory response, while the minipig model mirrors human anatomy. By integrating these complementary approaches, we aim to establish a comprehensive preclinical validation of the biomaterial’s ability to drive functional bone repair. Our goal is to demonstrate the capacity of this biomaterial to promote robust, vascularized, and innervated bone regeneration.

## Materials and methods

### The ELP-based composite matrix

The composite matrix used in this study was previously described by Mahmoudi *et al*. [34]. It consists of a recombinant ELP produced in *Escherichia coli*, purified *via* inverse transition cycling, and subsequently thioalkylated at methionine residues. The matrix also incorporates bioactive components, including cell adhesion peptides (IKVAV and YIGSR), a matrix metalloproteinase-2-–cleavable sequence (PVGLIG), and a calcium phosphate-nucleating peptide (SNA15). HA was synthesized using wet chemical precipitation at 40°C, with the pH maintained above 9, and then washed, freeze-dried, and sieved. Finally, 1.5% (w/v) ELP, 1.5% (w/v) peptides, and 2% (w/v) HA particles were mixed in polydimethylsiloxane (PDMS, Sylgard 184, Sigma-Aldrich cat. no. 761036) molds, frozen, and photo-cross-linked with Irgacure^®^ 2959 under UV light (#410896, CAS Number: 106797-53-9, Sigma-Aldrich). The resulting ELP-based composite matrices were rehydrated and freeze-dried for subsequent applications.

### Mandibular bone defects in rats

Bone defect implantations were performed bilaterally in 12-week-old female Wistar rats (Janvier Labs^®^, RjHan:WI) (*n* = 28). The animal experimentation protocol was approved by the local ethics committee (protocol #32463-202201261842498). Animals were acclimatized for 2 weeks prior to surgery. Buprenorphine was administered subcutaneously 20 min prior to surgery and during the subsequent two days. To create the mandibular defect, rats were first anesthetized in an induction chamber by inhalation of 4% isoflurane at 1.5 L/min, followed by maintenance anesthesia with 2% isoflurane at 0.9 L/min via a face mask throughout the duration of the surgery. The implantation sites were shaved and disinfected with antiseptic solution, the muscles were dissected to expose the mandibular bone, and a bilateral circular lesion was created by removing an entire 3.3-mm diameter bone disk using a trephine, producing a full-thickness defect in the lateral (ramus) region of the mandible (Supplementary Figure S1). This defect size corresponds to a critical-size mandibular defect, defined as a defect that does not undergo spontaneous healing, as established in craniofacial bone regeneration models. The defects were left empty (*n* = 18), or filled with either Collapat^®^ (*n* = 19) or with the ELP-based composite matrix (*n* = 19). Collapat^®^ (SYMATESE^®^ Biomaterial, France), a macroporous sponge composed of bovine-derived collagen proteins and synthetic HA granules, was used as a commercial control material. The postoperative course was uneventful. Rats were followed for 3 days postoperatively with analgesic treatment, and no deaths were observed.

### Mini-pig alveolar defect

The animal experimentation protocol in Göttingen minipigs, approved by the local ethics committee (protocol #2021-15-0201-00876) was conducted under good manufacturing practices conditions in the Ellegard Göttingen minipig facility, Dalmose, Denmark, as previously described by Imber *et al*. [35]. Briefly, bilateral extraction of eight teeth (the mandibular premolars and first molar) per animal were performed in four animals, and therefore creating extraction sockets in the alveolar bone. After a 3-month healing period, alveolar defects were created as follows: an incision was made in the edentulous region of the mandible, and a full-thickness flap was raised; the exposed ridge was gently flattened to achieve a uniform surface, and four cylindrical defects (4.2 mm in diameter × 6 mm in depth) were created in each hemimandible (Supplementary Figure S2). The ELP-based composite matrix, along with a xenograft matrix often used for such clinical application (Straumann^®^; S1-0210-050; batch B221371B) as commercial control, were implanted into these alveolar defects (Supplementary Figure S2). Soft tissues were then repositioned and closed with resorbable sutures. The postoperative course was uneventful. No deaths were observed. Two animals were euthanized 1-month postimplantation, and the remaining two after 2 months of implantation. Hemimandibles containing the defects were subsequently collected for histological processing. The xenograft matrix (Straumann^®^) is a sterile, bovine-derived granulated biomaterial (200–1000 µm) engineered for bone regeneration, widely used as a commercial reference material to closely replicate clinical conditions, as it reflects the standard of care employed by clinicians in patient treatment.

### X-ray microcomputed tomography

To assess new bone formation 1 week, 2 weeks, and 4 weeks after implantation, mandibular rat bone defects were analyzed using a microcomputed tomography (micro-CT) scanner (Quantum FX Caliper, Life Sciences^®^, Perkin Elmer, Waltham, MA) as described by Mahmoudi *et al*. [34] The region of interest (ROI) was defined as the volume of the initial bone defect, allowing quantification of the newly formed bone volume relative to the total sample volume (mineral volume/total volume=MV/TV).

### Histological and immunohistological analyses

After sacrificing rats in a CO_2_ chamber, the entire hemimandibles and surrounding tissues were collected and fixed for 24 h at room temperature (RT) in 4% (w/v) paraformaldehyde (PFA -Antigenfix^®^, DIAPATH). The tissue was decalcified using Microdec^®^ solution (DIAPATH, France) for at least 1 month, with the solution changed weekly. For processing of the minipig tissue, decalcification was performed for 6 months in percentage (w/v) EDTA at pH = 7.6, with the solution changed weekly. All samples were subsequently dehydrated and embedded in paraffin. Sections of 7 μm thickness were prepared using a microtome (Erpedia^®^ HM 340E). Before staining or immunolabeling, the slides were deparaffinized in three baths of OTTIX^®^ (DIAPATH) for 10 min each, and rehydrated by successive washes for 5 min with ethanol of decreasing concentration (100%, 95%, 70%).

Histological sections were stained with a modified Masson’s trichrome (where aniline blue is replaced by light green). Slides were incubated in three successive solutions: Hemalun (VWR, #1.09249.2500), a 1% (w/v) Fuchsin-Ponceau mixture (VWR/RAL, #313200-0025, RAL #316150-0025), and 1% (w/v) light green (Merck #1.15941.0025). After staining, sections were dehydrated in increasing concentrations of ethanol (70%, 95%, 100%) for 2 min each, followed by three 2-min baths of OTTIX^®^. Finally, slides were mounted in Diamount^®^ (MM France).

In parallel, histological sections were also processed for immunostaining. Briefly, after deparaffinization and rehydration, tissue sections were fixed in 4% (w/v) PFA for 15 min at RT, followed by a step of unmasking antigenic sites in PT Link [unmasking bath (low pH), Agilent Technologies, Inc.; Dako #DM829, K8005] for 20 min at 95°C. Samples were permeabilized in solutions of increasing concentrations of Triton diluted from a Triton X-100 (ACROS Organics, #215680010) stock solution in Dulbecco’s Phosphate Buffered Saline 1X (DPBS) prepared from a DPBS 10× solution (Gibco, #14200-067): 0.025% for 5 min and 0.5% Triton for 30 min at RT. The samples were then incubated in a blocking solution of Triton 0.1% diluted in DPBS 1×, with 2% of Bovine Serum Albumin 10× (VWR, #422361 V) and 5% of normal goat serum 100× (Sigma-Aldrich, #S26-100 mL) for 1.5 h at RT. Then, the primary antibody solutions were prepared in the blocking solution—anti-NeuroFilament (Abcam, #ab8135, 1:1000) and anti-CD11b (Invitrogen, #PA5-79532, 1:100)—and deposited on the samples overnight at 4°C, in the dark. After rinsing with DPBS 1×, samples were incubated in a solution of Alexa Fluor 568-conjugated anti-rabbit IgG secondary antibody (Invitrogen, #A-10042) diluted at 1:500 in DPBS 1× for 1.5 h at RT in the dark. A final incubation of 20 min with DAPI (Thermo Scientific, Germany, #62248) diluted at 1:1000 in DPBS 1× was performed at RT. Slides were mounted using AquaPolymount (Polysciences, VWR, #18606-20).

### Whole-mount immunostaining

Whole-mount immunolabeling was adapted from a previous protocol [36] as follows. Rats were anesthetized, and then perfused intracardially with DPBS 1× followed by 4% PFA, and the entire hemimandibles and surrounding tissues were collected and immersed in 4% PFA for 24 h at 4°C. Decalcification was performed in 1% EDTA at RT for 2 weeks with several solution changes. Next, tissues were dehydrated in successive methanol solutions (20%, 40%, 60%, 80%, 100%) for 1 h each, and bleached overnight at 4°C in 6% H_2_O_2_/methanol. After rehydration (in successive methanol solutions from 100% to 20%), samples were washed twice in PBS-GSTT (PBS with 0.2% gelatin, 0.1% saponin, 0.5% Triton X-100, 0.01% Thimerosal) and blocked in the same solution for 2 days at 37°C. Primary antibodies—anti-β3-Tubulin (Abcam, #ab18207, 1:1000), anti-Endomucin (Santa Cruz, #sc65495, 1:100), anti-MECA32 (BD Pharmingen, #550563, 1:10), and anti-Podocalyxin (R&D, #MAB1556, 1:1000)—were incubated for 2 weeks at 37°C. After washing, secondary antibodies were incubated for 6 days at 37°C, followed by washes in PBS-GSTT at RT. Tissues were cleared following the iDISCO+ protocol [37]. Briefly, samples were dehydrated in methanol (20%, 40%, 60%, 80%, 100%) for 1 h each, then delipidated for 3 h at RT in a dichloromethane/methanol mixture (1:3). Finally, samples were cleared overnight in dibenzyl ether (Sigma, #108014) at RT.

### Image acquisition and processing

Bright-field image acquisitions were performed using a slide-scanning microscope equipped with a TDI 3-CCD camera and Nanozoomer Digital Pathology^®^ software (version 2.0, Hamamatsu Photonics, Japan). Histological sections processed for fluorescent immunostaining were imaged with a confocal laser scanning microscope (SP8, Leica^®^ Microsystems or Stellaris, Leica^®^ Microsystems). Whole-mount fluorescently immunostained samples were imaged using a light-sheet fluorescence ultramicroscope (LaVision BioTec Ultramicroscope II) with ImspectorPro^®^ software (LaVision BioTec, Bielefeld, Germany).

Colorimetric bone tissue analyses of the lesions in rats and minipigs were performed using ImageJ (version 1.53t, National Institutes of Health, Bethesda, MD, USA) and QuPath software (version 0.5.0, University of Edinburgh, Edinburgh, UK), respectively, based on manual annotations. An ROI corresponding to the entire lesion area was defined, and the bone tissue area was manually identified.

Blood vessels were manually quantified using NDPView^®^ software (version 2.9.29, Hamamatsu Photonics, Hamamatsu, Japan). An ROI corresponding to the entire defect area was defined for each sample, and the number of blood vessels was counted on histological sections.

Regarding histological sections processed for fluorescent immunostaining, quantification analyses were performed with the IMARIS^®^ software (version 9.0.1, Bitplane, Zurich, Switzerland). ROIs corresponding to the total lesion area were manually defined based on tissue autofluorescence (Surface plugin). The area occupied by nerves was determined using the Surface plugin, and the number of CD11b positive cells was measured with the Spots plugin. The parameters used to quantify the samples containing the ELP biomaterial were adjusted to take into account the autofluorescence of the ELP polymer.

### Statistical analyses

All statistical analyses were performed with GraphPad Prism^®^ (version 8.0.2, GraphPad Software, San Diego, CA, USA). As the data did not meet the assumptions of normality, statistical significance was assessed using the Kruskal–Wallis nonparametric test, followed by appropriate *post hoc* tests for multiple comparisons. Results are expressed as mean values ± standard deviation.

## Results

### ELP-based matrices enhance mineral accumulation in mandibular bone defects

**Figure 1:**
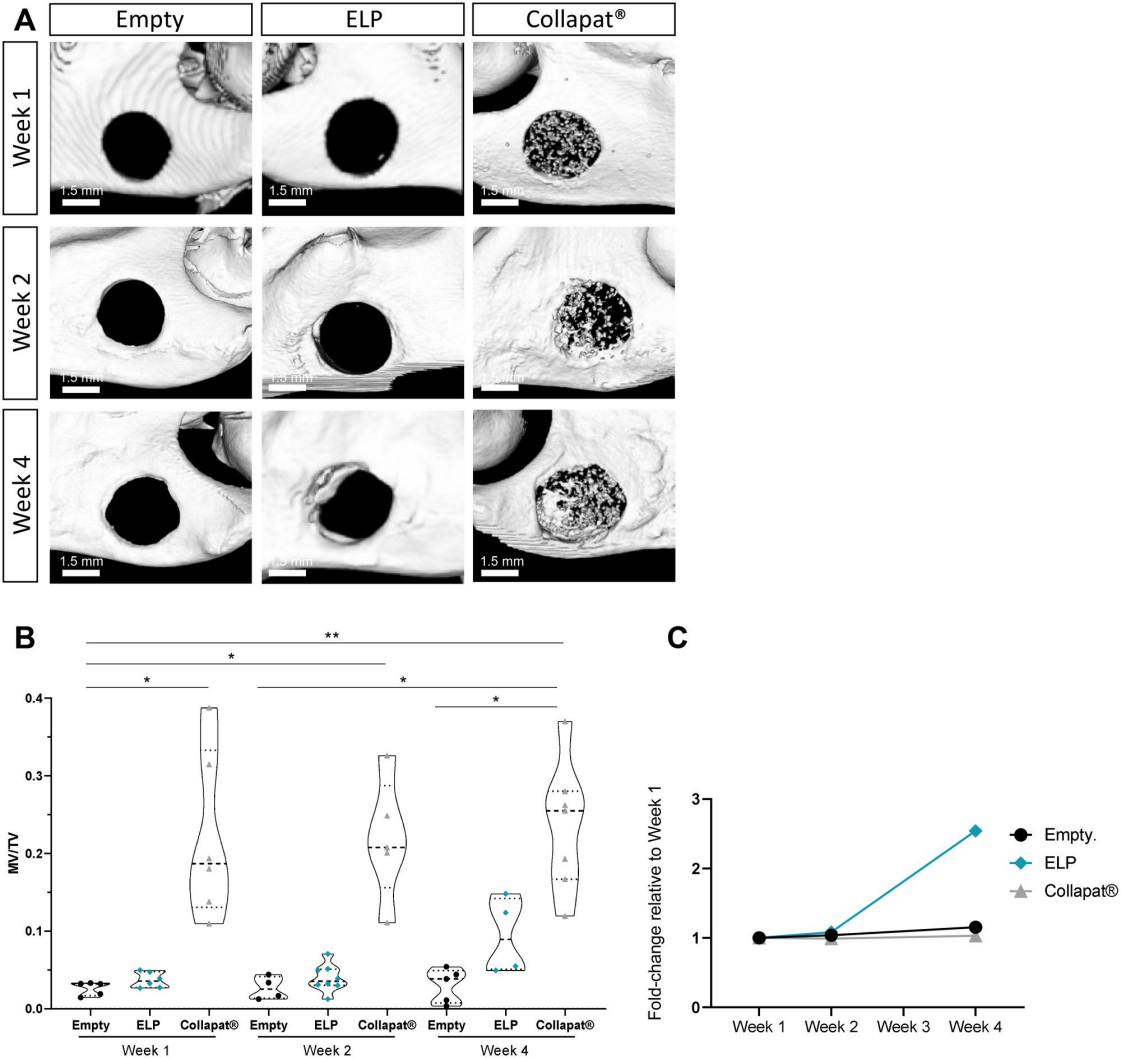
Mineralization potential of matrices after implantation in the rat mandible lesion model. (**A**) Representative images from 3D reconstructions of micro-CT acquisition of the implantation site in the mandible model. Mineralization was monitored at 1 week, 2 weeks, and 4 weeks after implantation in the three groups: lesion left empty, lesion filled with ELPs matrices, and lesion filled with the commercial product Collapat^®^. Scale bar: 1.5 mm. (**B**) Mineral volume/total volume (MV/TV) ratio was measured from 3D reconstructed micro-CT images presented in (**A**). The measurements were carried out using Microview^®^ software for the three groups and for all implantation times. A Kruskal–Wallis multiple comparison test was performed. Data are represented as mean ± SD (*P < 0.05 and **P < 0.01, respectively), *n* = 6 rats per condition. (**C**) Increase in mineralized volume between week 1 and week 4. Data for each condition were normalized to the mineral volume/total volume (MV/TV) ratio measured at week 1, and are presented as the mean fold-change relative to baseline (week 1).

To assess the osteogenic potential of the composite matrices, the ELP-based matrix and the Collapat^®^ commercial control were implanted into a rat mandibular defect model, while unfilled defects served as negative controls. Total mineral content was monitored using micro-CT at different time points (weeks 1, 2, and 4) (Figure 1). Representative images for each condition and time point, highlighting the mandibular defect site, are shown in panel A, while the corresponding quantitative analysis is presented in panel B.

Micro-CT quantitative analysis of the mineral volume fraction (MV/TV) revealed distinct mineralization profiles among the groups (Figure 1B). In the empty control group, no significant mineralization was observed throughout the time period studied, indicating the inability of spontaneous healing to occur in this model. The ELP matrix group showed no visible mineralization at week 1, but exhibited partial peripheral reconstruction of the defect by week 4, translated by the MV/TV ratio which remained relatively stable between week 1 (0.037 ± 0.010) and week 2 (0.040 ± 0.018), with a slight increase observed by week 4 (0.094 ± 0.049), suggesting progressive osteogenic activity. In contrast, the Collapat^®^ group, due to its inherently mineral composition, demonstrated consistently elevated mineralized content from week 1 to week 4, with values remaining relatively unchanged over time. This becomes evident when normalizing the MV/TV values to those obtained at week 1 for each condition (Figure 1C). The data reveal that both the unfilled defects and the Collapat^®^ group exhibit similar mineralization kinetics, with no substantial increase over time. In contrast, the composite ELP matrices display a markedly higher mineral content after 1 month of implantation, highlighting their progressive osteogenic response.

### ELP matrices are highly biocompatible and elicit only negligible inflammation

**Figure 2:**
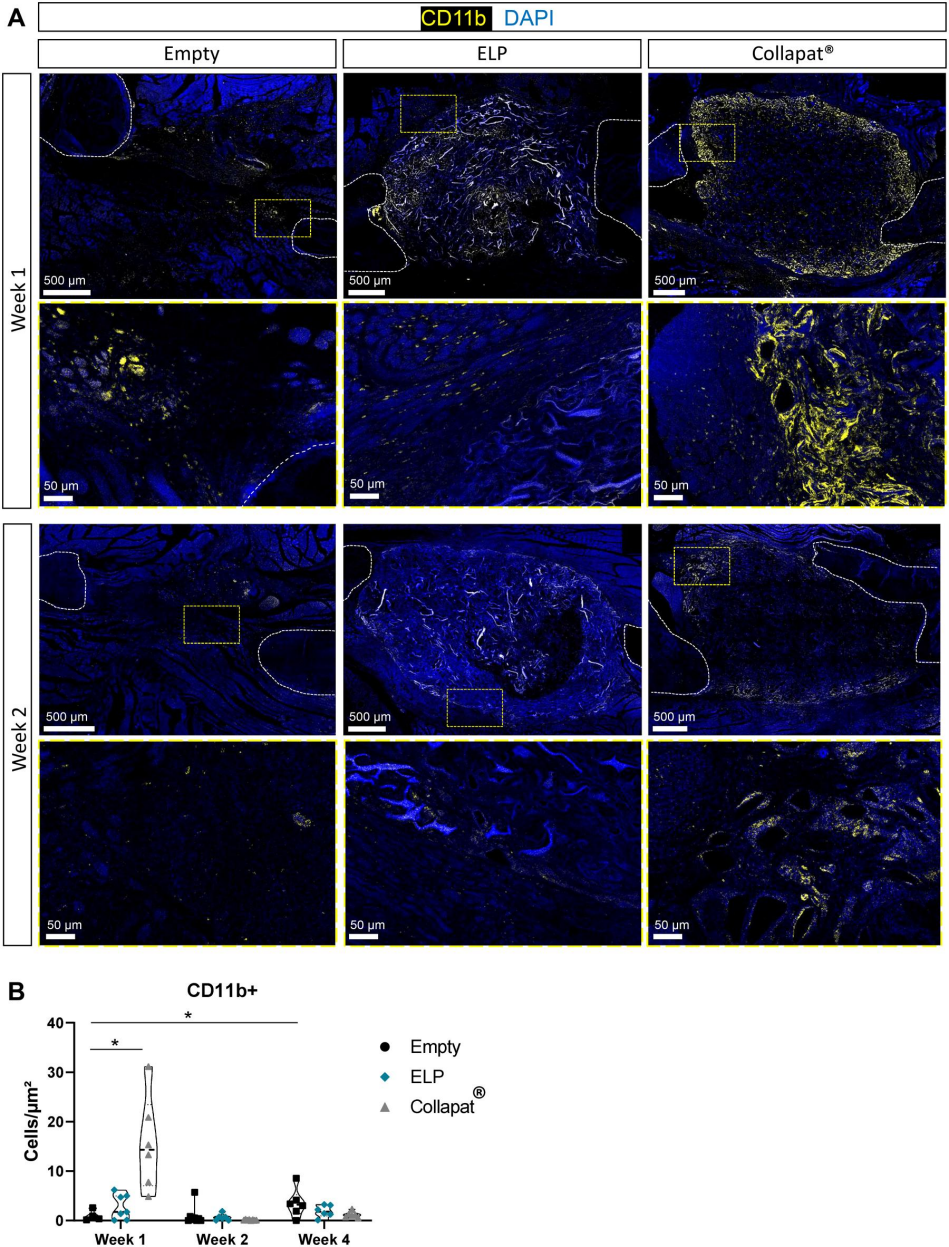
Inflammatory profile of matrices after implantation in the rat mandible lesion model. (**A)** Immunostaining of the CD11b marker (yellow) on histological sections of bone lesions filled with the ELP composite matrix, Collapat^®^ or empty groups after 1 week and 2 weeks of implantation. Sections representing the whole lesion shown on top, and magnification which corresponds to the yellow dotted box on the images is represented on the bottom. Cell nuclei were labeled with DAPI (blue). The white lines delimit the area corresponding to the bone tissue. Scale bars: 500 μm, 50 μm in insets. (**B)** Graph showing the number of CD11b+ cells in the matrices and at their periphery. A Kruskal–Wallis multiple comparison test was performed. Data represent the mean ± SD (*P < 0.05). *n* = *x*; *y*, where *x* indicates the number of rats and *y* the number of sections analyzed for each condition: at week 1: *n* = 4; 4 for empty, 3; 7 for ELP, and 3; 6 for Collapat^®^. At week 2: *n* = 4; 8 for empty, 3; 6 for ELP, and 4; 8 for Collapat^®^. At week 4: *n* = 3; 6 for all groups.

The early inflammatory response was evaluated by assessing the presence of CD11b^+^ immune cells by immunofluorescence staining after 1 and 2 weeks of implantation (Figure 2A). CD11b^+^ immune cells are depicted in yellow, and nuclei are counterstained with DAPI (blue). Cell density then was quantified per square micrometer (Figure 2B).

At week 1, a significantly higher density of CD11b^+^ cells was observed in the Collapat^®^ group (15.55 ± 9.10 cells/µm^2^) compared to both the ELP (2.74 ± 2.45 cells/µm^2^) and empty groups, with CD11b^+^ cells predominantly localized at the periphery of the Collapat^®^ implant. In contrast, the ELP group exhibited minimal immune cell infiltration, with a sparse and diffuse CD11b^+^ signal, comparable to what was observed with the empty defect. This suggests that the ELP matrix induced a milder early immune response than Collapat^®^.

By week 2, a marked reduction in CD11b^+^ cells was observed in both ELP (0.64 ± 0.65 cells/µm^2^) and Collapat^®^ (0.13 ± 0.08 cells/µm^2^), confirming the rapid resolution of acute inflammation. CD11b^+^ cell levels in the empty group remained similarly low (0.96 ± 1.91 cells/µm^2^).

At week 4, low CD11b^+^ cell densities persisted across all groups, with slightly higher values in the empty group (3.49 ± 2.74 cells/µm^2^) compared to ELP (1.88 ± 1.15 cells/µm^2^) and Collapat^®^ (1.20 ± 0.60 cells/µm^2^).

The persistently low immune cell density in the ELP group reinforces its biocompatibility and supports its potential for promoting constructive tissue remodeling in a low-inflammatory microenvironment.

### ELP-based composites promote osteoconduction in mandibular bone defects

**Figure 3:**
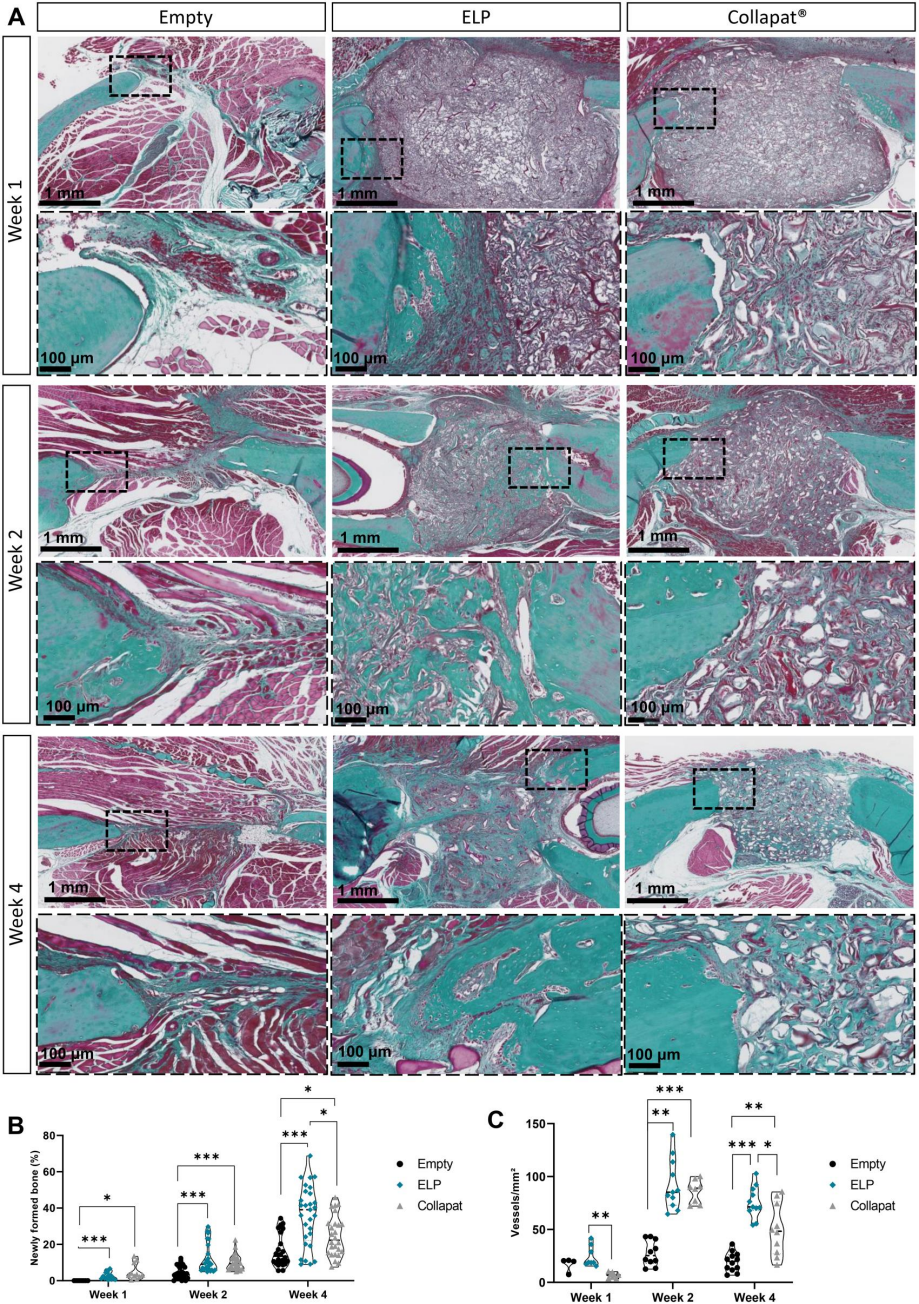
Osteogenic and angiogenic potential of matrices after implantation in the rat mandible lesion model. (**A**) Representative images of Masson’s trichrome staining on cross-sections of the bone lesions, after 1 week, 2 weeks, and 4 weeks of implantation, for the three groups: lesion left empty, lesion filled with ELPs matrices, and lesion filled with Collapat^®^. For each time point, sections representing the whole lesion are shown on top, and magnification which corresponds to the black dotted box on the images is represented on the bottom. Scale bars: 1 mm, 100 μm at higher magnifications. (**B**) Graph representing the percentage of newly formed bone for each of the groups. A Kruskal–Wallis multiple comparison test was used. Data are represented as mean ± SD (*P < 0.05 and ***P < 0.001, respectively). *n *= *x*; *y* where *x* indicates the number of rats and *y* the number of sections analyzed for each condition. At week 1 = 3; 16 for empty, 3; 17 for ELP, 3; 18 for Collapat^®^. At week 2 = 4; 22 for empty, 4; 27 for ELP, 4; 25 for Collapat^®^. At week 4 = 4; 24 for empty, 4; 28 for ELP and 4; 29 for Collapat^®^. (**C**) Graph representing the ratio of blood vessels per cubic millimeter for each of the groups. A Kruskal–Wallis multiple comparison test was used. Data are represented as mean ± SD (*P < 0.05, **P < 0.01, ***P < 0.001, respectively). *n* = *x*; *y*, where *x* indicates the number of rats and *y* the number of sections analyzed for each condition. At week 1 = 3; 4 for empty, 3; 8 for ELP, and 3; 8 for Collapat^®^. At week 2 = 4; 10 for empty, 4; 11 for ELP, 4; 8 for Collapat^®^. At week 4 = 4; 12 for empty, 4; 11 for ELP, and 4; 10 for Collapat^®^.

Histological analysis was performed after 1, 2, and 4 weeks of implantation to assess tissue integration and bone formation. Masson’s trichrome staining (Figure 3A) was used to differentiate newly formed osteoid tissue from surrounding connective tissue.

In the negative control (empty group), the lesion was progressively filled with fibrous collagenous tissue, with limited osteoid formation restricted to the defect’s margins. Osteoid tissue only became apparent after 4 weeks of implantation, suggesting slow endogenous bone regeneration.

In contrast, ELP matrices triggered progressive osteoid tissue deposition from the periphery toward the center of the defect, indicating effective osteoconduction. Histological analysis revealed good integration, with numerous cellular nuclei present within the matrix, indicative of effective cellular colonization (Supplementary Figure S3). Importantly, no signs of fibrotic encapsulation were observed, confirming the biocompatibility of the ELP-based scaffolds. Notably, the presence of osteocyte-like cells embedded in the matrix from week 2 onward suggests that early bone remodeling was initiated, as evidenced by the presence of lamellar bone tissue within the ELP group (Supplementary Figure S3).

In the Collapat^®^ group, scattered osteoid formation was observed, predominantly at the defect margins in direct contact with native bone. Unlike the ELP matrices, bone ingrowth appeared less homogeneously distributed, suggesting a more localized osteogenic response.

Quantification of bone formation (Figure 3B) confirmed these histological trends. At week 1, no bone formation was detected in the empty group, while low levels were observed in the ELP (2.77 ± 2.04%) and Collapat^®^ (4.90 ± 4.68%) groups. At week 2, a moderate increase was noted in all groups, but the most pronounced differences appeared at week 4. The ELP group reached 35.79 ± 16.85% of newly formed bone, significantly higher than Collapat^®^ (24.44 ± 11.24%) or the empty group (16.33 ± 9.12%) (*P < 0.05 to ***P < 0.001), suggesting the osteoconductive potential of the ELP-based matrix.

### Composite matrices enhance vascularization and nerve ingrowth

In parallel, vascular density was assessed by quantifying the number of blood vessels per square millimeter of tissue within the lesions, based on their morphological characteristics and the presence of red blood cells (Figure 3C). A considerable angiogenic response was observed at week 2, with both the ELP and Collapat^®^ groups showing significantly higher vascular density (92.5± 24.0 and 86.0 ± 10.9 vessels/mm^2^, respectively) compared to the empty group (27.6 ± 11.3 vessels/mm^2^). This response suggests active early vascular infiltration induced by both materials.

However, by week 4, a decrease in vascular density was observed in both groups. Despite this reduction, ELP-treated defects maintained the highest vascular density (75.6 ± 14.9 vessels/mm^2^), compared to Collapat^®^ (49.6 ± 24.5 vessels/mm^2^) and the empty group (19.5 ± 9.0 vessels/mm^2^). This reduction may be attributed to the remodeling of initially abundant capillary networks into fewer but larger and more mature vessels, a process commonly observed during vascular maturation. This hypothesis is further supported by the improved tissue organization and the presence of larger perfused structures observed in histological sections at week 4 (Supplementary Figure S3).

**Figure 4:**
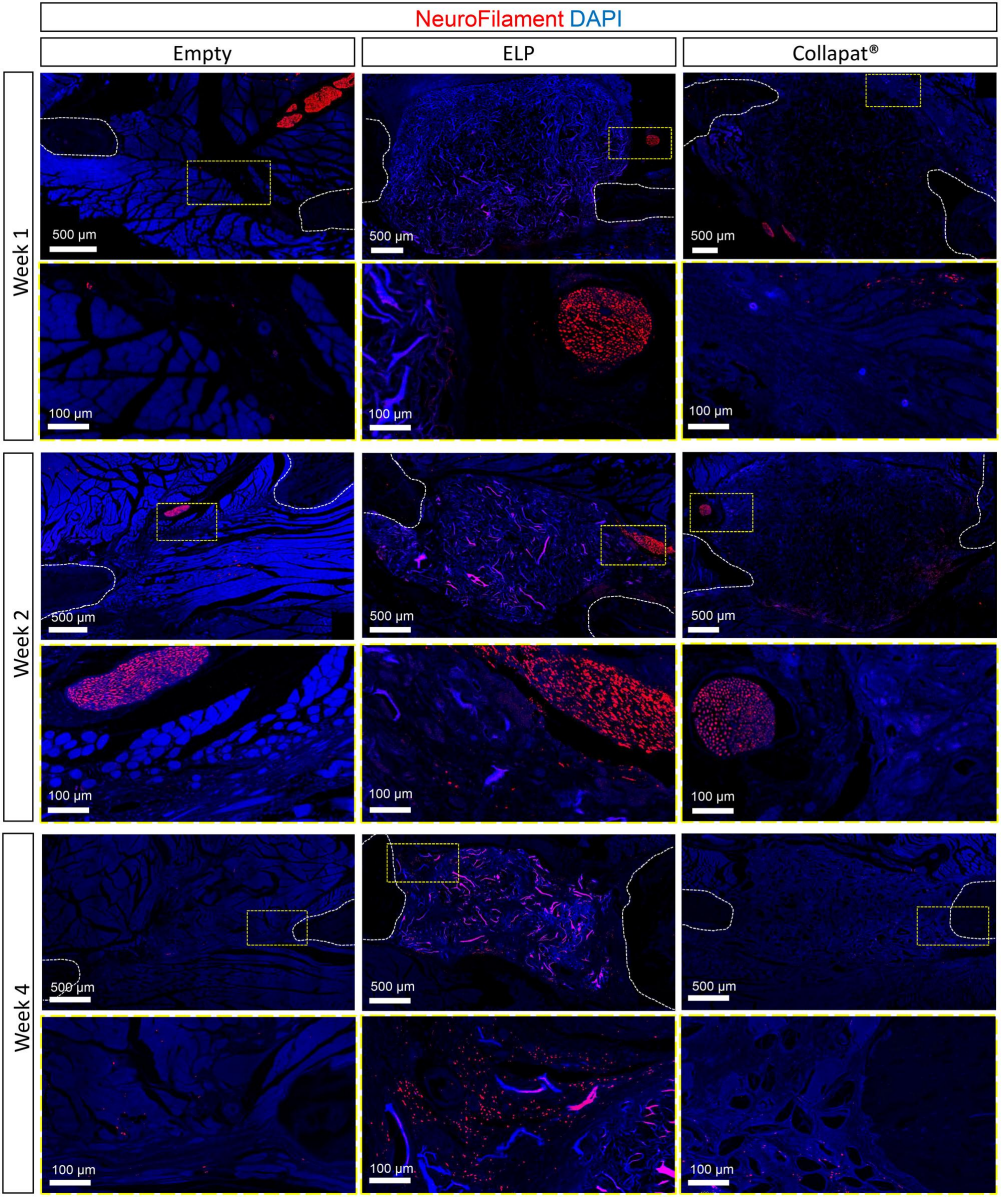
Innervation of matrices after implantation in the rat mandible lesion model. Immunostaining on histological sections of bone lesions filled with the ELP composite matrix, Collapat^®^ or empty groups after 1 week, 2 weeks, and 4 weeks of implantation highlights the neuronal network evidenced by NeuroFilament immunostaining (in red) for each time point and for each group, sections representing the whole lesion shown on top on each panel, and magnification which corresponds to the yellow dotted box on the images is represented on the bottom. Cell nuclei were labeled with DAPI (blue). The white lines delimit the area corresponding to the native bone tissue. Scale bars: 500 μm, 100 μm in insets.

To assess innervation within the mandibular defect, neurofilament (NF^+^) immunofluorescence staining was performed at weeks 1, 2, and 4 (Figure 4). At week 1, all groups exhibited preexistent Neurofilament^+^ nerve fibers that appeared predominantly sparsely distributed at the periphery of the lesion, and distant from the central defect area.

By week 2, evidence of innervation was observed in the ELP group, with numerous smaller axonal fibers sprouting in the vicinity of the preexistent nerve bundles. In contrast, both the empty defect and the Collapat^®^ group displayed well-defined preexistent nerve bundles at the periphery of the defect, but no neurofilament^+^ axonal fibers were observed within the defect area.

At week 4, the ELP matrix continued to support axonal ingrowth, with fibers extending in the central region of the defect in close contact with the scaffold. Regarding the empty and Collapat^®^ groups, imaging revealed a sparse distribution of neuronal fibers within the lesion site, indicating limited innervation across these conditions.

**Figure 5:**
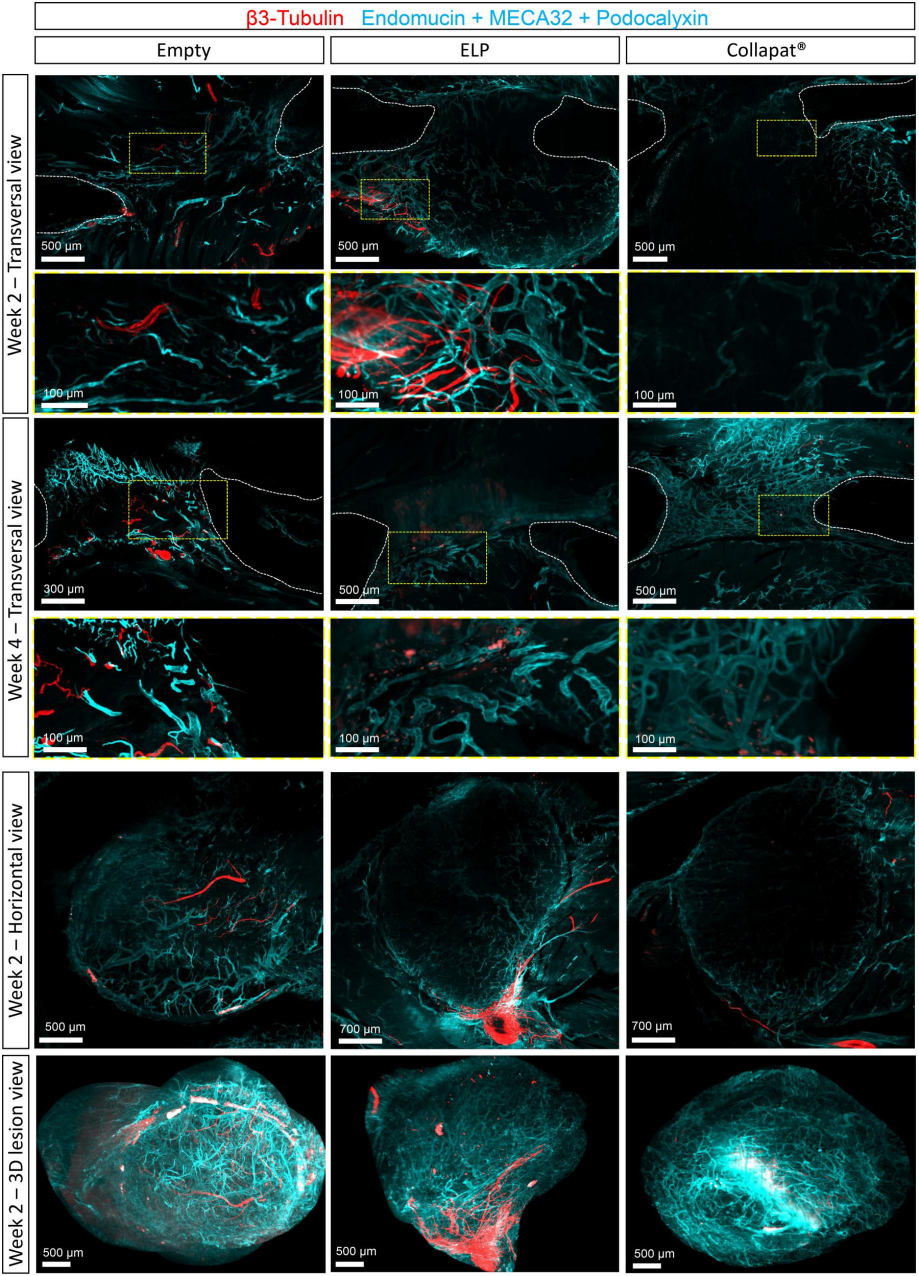
3D Reconstructions of blood vessels and nerve fibers immunostaining in matrices after implantation in the rat mandible lesion model. 3D reconstructions of the entire mandible lesions were imaged by light-sheet microscopy after 2 weeks and 4 weeks of implantation in the rat mandible lesion model. β3-Tubulin staining is shown in red to evidence the neural network. In cyan the staining of Endomucin, Podocalyxin, and MECA32 to reveal the vascular network. The mandible bone lesions are presented in transversal view, horizontal view, and 3D reconstruction of the entire lesion from top panel to bottom, respectively. Sections representing the whole lesion shown on top on each panel, and magnification which corresponds to the yellow dotted box on the images is represented on the bottom. The white lines delimit the area corresponding to the bone tissue. Scale bars: 300/500/700 μm, 100 μm in insets.

Due to the heterogeneous distribution of neuronal fibers at the tissue periphery and within the lesion site, 2D histological sections were insufficient for reliable quantification of innervation in the defect region. To overcome this limitation, whole-mount cleared tissue imaging was employed, enabling volumetric visualization of the neurovascular architecture across the entire defect area (Figure 5). Axons (β3-Tubulin immunostaining, red color) and blood vessels (Endomucin, MECA32, and Podocalyxin immunostainings, blue color) were visualized using light sheet microscopy.

At weeks 2 and 4, whole-mount imaging revealed vascular structures in all three groups, as previously described by 2D histology (Figure 3). In the ELP group, densely interconnected blood vessels were distributed throughout the scaffold, indicating effective vascular infiltration into the defect. Regarding innervation, whole-mount imaging at week 2 revealed β3-Tubulin^+^ nerve bundles extending from the periphery toward the core of the ELP scaffold, exhibiting a dense, branched network in close association with the vascular architecture. This suggests that the ELP scaffold may provide a more permissive environment for peripheral nerve extension within the material. The empty group also displayed centrally located β3-Tubulin^+^ bundles, though with noticeably less arborization than the ELP group. In contrast, the Collapat^®^ group showed sparse neuronal fiber presence, primarily limited to the lesion margins. By week 4, ELP-filled defects contained abundant β3-Tubulin^+^ bundles distributed throughout the scaffold, penetrating deep into the matrix. Conversely, Collapat^®^-treated defects continued to exhibit scattered neuronal structures, largely confined to the defect periphery.

3D reconstructions of the total defect area confirmed the presence of vascular structures and sprouting nerve bundles within the ELP scaffold, while empty lesions showed less organized nerve bundles and defects filled with Collapat^®^ presented limited presence of nerves overall. Due to limited resolution of the whole-mount imaging, only large nerve bundles could be observed in the cleared tissue (Supplementary Videos S1–S6).

These findings confirm that the ELP matrix promotes both vascular and neuronal integration, crucial for bone regeneration, while the Collapat^®^ scaffold exhibits limited neurovascular support, potentially impacting its regenerative potential.

### Osteoconductive potential of ELP-based matrices in alveolar bone defects of minipigs

**Figure 6:**
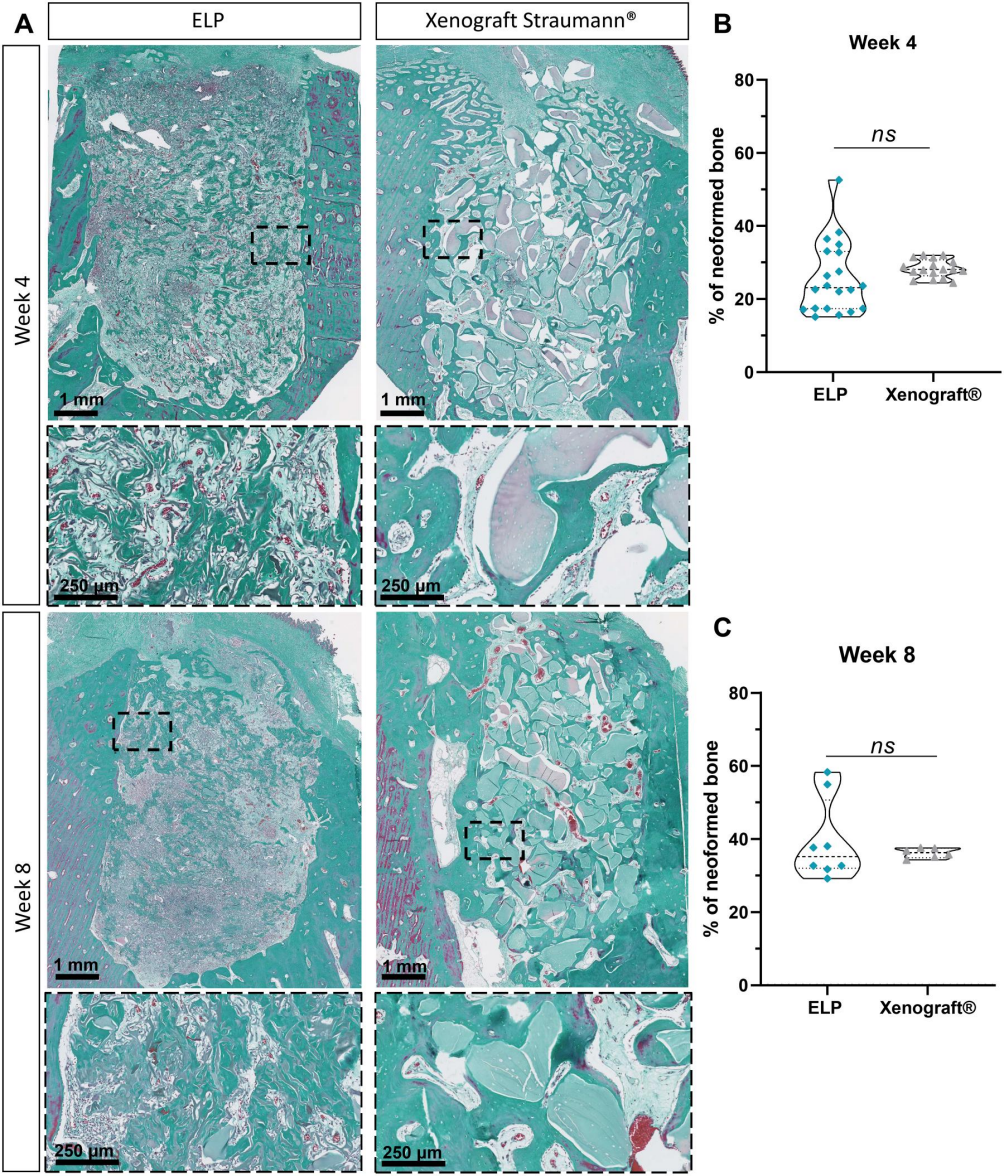
Osteogenic potential of matrices after implantation in the mini-pig alveolar lesion model. (**A**) Representative images of Masson’s trichrome staining on alveolar lesion sections, 4 weeks and 8 weeks after implantation of the ELP composite matrix and the Xenograft Straumann^®^ commercial xenograft granules. For each time point, sections representing the whole lesion are shown on top of the panel, and magnification which corresponds to the black dotted box on the images is represented on the bottom. Scale bars: 1 mm, 250 μm at higher magnifications. (**B**) Graph representing the percentage of newly formed bone for each of the groups, 4 weeks after implantation. A Kruskal–Wallis multiple comparison test was used. Data are represented as mean ± SD (ns, nonsignificant). *n* = *x*; *y*, where *x* indicates the number of lesions and *y* the number of sections analyzed for each condition: for ELP = 6; 20 and for Xenograft Straumann^®^=5; 17. (**C**) Graph representing the percentage of newly formed bone for each of the groups, 8 weeks after implantation. A Kruskal–Wallis multiple comparison test was used. Data are represented as mean ± SD (ns, nonsignificant). *n* = *x*; *y*, where *x* indicates the number of lesions and *y* the number of sections analyzed for each condition: for ELP = 4; 8 and for Xenograft Straumann^®^=3; 6.

Building on the promising results observed in the rat mandibular defect model, we next evaluated the translational potential of the ELP matrix in a clinically relevant large animal model. To this end, we assessed its capacity to promote alveolar bone regeneration in minipigs at weeks 4 and 8, and compared its performance to a commercially available positive control, generally used in this clinical context, the Xenograft from Straumann^®^. Histological analysis for both groups (tested material and Xenograft) and for both time points (weeks 4 and 8) was performed using Masson’s trichrome staining to evaluate osteoid deposition, tissue organization, and scaffold integration (Figure 6A).

Quantitative analysis of the newly formed bone (Figure 6B and C) showed similar percentages of neoformed bone with the ELP and xenograft samples, with no statistically significant difference between them (Figure 6B).

By week 8, bone formation had progressed in both groups, yet no significant difference in the percentage of mineralized tissue was detected between ELP and xenograft (Figure 6C).

However, even though no differences were measured in the total amount of newly formed bone tissue, histology analyses revealed major differences in the tissue organization. By week 4, robust cellular infiltration was observed throughout the scaffold in the ELP matrix group, particularly in the central regions of the defect (Figure 6A). These areas displayed active osteoid deposition, numerous blood vessels perfusing the tissue, along with zones of matrix remodeling (Supplementary Figure S4), indicative of progressive tissue integration and early bone formation. Conversely, in the xenograft-treated defects, osteoid accumulation was primarily restricted to the lesion periphery, in close proximity to the native bone. The central portion of the defect remained largely occupied by unresorbed scaffold granules, with few blood vessels in the center of the lesion, and evidence of slower remodeling activity.

By week 8, the differences in regenerative outcomes between the groups became even more pronounced. In the ELP matrix-treated defects, extensive osteoid deposition was observed throughout the lesion site, including deep within the scaffold. The osteoid appeared well organized, with lamellar-like structures (Supplementary Figure S4). Numerous blood vessels, both large and small, were present across the defect, often in close proximity to osteogenic regions, suggesting a coordinated vascularized bone regeneration process. Signs of ongoing remodeling, including osteoblast-lined surfaces, were also evident. In contrast, defects treated with the xenograft material (Straumann^®^ Xenograft) displayed reduced tissue infiltration into the scaffold core. Osteoid was largely confined to the defect margins adjacent to native bone and in the close periphery of the material’s granules. The interior of the lesion remained populated by unresorbed xenograft granules, with limited evidence of active bone remodeling. Collectively, these findings underscore the superior regenerative environment provided by the ELP composite.

## Discussion

Regenerating craniofacial bone defects requires more than simply filling a structural void; it demands a coordinated orchestration of osteogenesis, vascularization, and innervation to restore full tissue functionality [38]. Our study demonstrates that a bioinspired ELP-based composite matrix functionalized with bioactive peptides and hydroxyapatite particles enables this coordinated regeneration in both rat and minipig models, without the use of exogenous cells or growth factors. It fosters robust bone formation, tempers inflammatory responses, and supports the concurrent ingrowth of blood vessels and nerve fibers; essential drivers of durable, functional regeneration [39, 40]. These results position this ELP-based scaffold as a promising tool for clinical bone repair compared to two different commercial materials tested in this paper Collapat^®^ or Xenograft Straumann^®^. Indeed, each animal model required a control material appropriate to its scale, surgical context, and experimental setting. In the rat model, we used Collapat^®^, a microporous composite material composed of bovine-derived collagen proteins and synthetic HA granules. This composite shares structural similarities with our ELP-based matrix and is commonly used to fill small bone defects in maxillofacial surgery and dentistry. In the minipig model, we used the xenograft Straumann^®^, which is also a bovine bone-derived mineral matrix but lacks an organic phase. It is widely used as a scaffold to support guided bone regeneration in oral surgery applications in large animals. Overall, each control material was selected to provide a clinically relevant and meaningful benchmark within the specific anatomical and experimental context of the corresponding animal model.

While these commercial materials are commonly used in clinical settings, they often fail to support simultaneous neurovascular integration. In clinical applications, biomaterials must preserve defect structure and support tissue regeneration in a load-bearing and immune-sensitive environment without a high level of inflammation. Our ELP matrix achieved this balance, integrating seamlessly with host tissues while promoting neurovascular regeneration without requiring exogenous cells or growth factors. This contrasts with conventional strategies that depend on mesenchymal stem cell transplantation or the use of growth factors such as bone morphogenetic protein 2 (BMP-2). Prior studies using BMP-2–releasing scaffolds have reported comparable bone fill percentages (30%-40%) [41], but with increased risks of ectopic bone formation, inflammation, and regulatory burden [42]. Our ELP matrix reached similar levels of bone formation (36% in rats and 35% in minipigs), without requiring exogenous growth factors, and with significantly reduced inflammation. These findings highlight the potential of intrinsic peptide signaling over growth factor delivery, such as BMP-2 or VEGF [43], which in addition pose challenges in terms of cost, regulation, and safety. By delivering intrinsic bioactivity through its peptide-functionalized design, the ELP matrix offers a safer, more scalable, and clinically relevant alternative.

The modular design of the engineered ELP matrix played a key role in supporting coordinated tissue regeneration. The incorporation of IKVAV and YIGSR peptides likely enhanced early cell adhesion and neurite outgrowth, while the MMP2-cleavable PVGLIG sequence enabled scaffold remodeling synchronized with tissue infiltration. This design enabled the simultaneous presence of β3-Tubulin^+^ nerve fibers, Endomucin^+^ vasculature, and osteoid tissue within the same regions of the defect, suggesting a spatial and temporal coupling between neural, vascular, and osteogenic processes.

Such integration may be facilitated by the permissive, low-inflammatory environment created by the engineered ELP matrix. Our *in vivo* results revealed a dynamic interface between host tissue and scaffold, characterized by early cellular infiltration, osteoid bridging, and vascular integration. This bioactivity is consistent with previous work on elastin-like polypeptide matrices in regenerative medicine, where controlled degradation and peptide signaling enhanced tissue remodeling [43, 44].

Controlling the immune response is essential for long-term graft acceptance, especially in craniofacial sites where inflammation can impair both hard and soft tissue integration [45]. Here, the ELP matrix exhibited strong immunotolerance, evidenced by minimal CD11b^+^ cell infiltration and absence of fibrotic encapsulation, unlike the inflammatory profile triggered by the bovine-derived Collapat^®^ scaffold (Figure 2).

This favorable profile likely reflects the animal-free, low-immunogenic composition of the ELP matrix, which avoids common complications linked to xenografts [46]. These observations are consistent with previous findings showing that prolonged myeloid cell recruitment around xenogenic or mineral-dense materials compromises tissue regeneration and favors fibrosis [47]. In our model, the absence of fibrous encapsulation in ELP-treated sites, compared to the intense early infiltration and delayed remodeling observed in Collapat^®^ implants, aligns with prior findings on xenogenic materials [48].

A major challenge in scaffold design is synchronizing material degradation with tissue regeneration. Mineral-based scaffolds such as Collapat^®^ often degrade slowly and passively, failing to adapt to tissue dynamics. By contrast, the ELP matrix integrates a PVGLIG sequence cleavable by MMP2, enabling cell-driven, environment-responsive degradation. This controlled remodeling was evident in our study, where the ELP matrix degraded in parallel with new tissue formation, whereas the Collapat^®^ and Straumann^®^ materials remained largely intact. These findings are consistent with our previous work using a similar ELP-based system, where MMP2-mediated degradation facilitated scaffold remodeling in concert with osteogenic progression [34]. An additional benefit of the ELP matrix is its radio-transparency, which facilitates high-resolution, noninvasive monitoring of healing *via* micro-CT. Unlike Collapat^®^, whose strong calcium phosphate signal masked new bone formation, the ELP matrix allowed clear visualization of mineral accrual over time (Figure 1A and B). Between weeks 2 and 4, bone volume nearly tripled in ELP-treated defects (Figure 1C), reflecting true osteogenesis.

To validate translational relevance, we assessed the performance of the ELP matrix in two complementary preclinical models: the rat mandibular defect and the minipig alveolar defect. These models differ in scale, healing dynamics, and translational applicability, offering a robust evaluation of scaffold efficacy across anatomical and physiological contexts. In both models, the ELP matrix conformed well to the defect geometry, with its adjustable volume ensuring optimal fit and close tissue contact. Its ease of use during surgery, whatever the preclinical models, as shown in Supplementary Figures S1 and S2, enabled precise placement and probably contributed to enhanced integration at the host–implant interface.

In rats, the ELP matrix promoted centripetal bone formation, with osteoid deposition extending from the defect margins toward the center by week 4 (Figures 1A and 3). This regenerative pattern suggests an active role of this matrix in guiding endogenous cells toward the lesion core, possibly through the establishment of a pro-regenerative gradient within the matrix. Collapat^®^ implants showed limited bone growth, mostly restricted to the periphery of the defect, with irregular and discontinuous integration into the host bone tissue, suggesting a less coordinated regenerative response. These differences were substantiated quantitatively, with 36% bone filled in the ELP group compared to 24% in Collapat^®^ and only 16% in empty defects (Figure 3B). In addition to osteogenesis, the ELP matrix also demonstrated a strong ability to support angiogenesis (Figures 3C and 5) and innervation (Figures 4 and 5) compared to Collapat^®^. Vessel density (Figure 3C) and β3-Tubulin^+^ axonal presence (Figure 4) were markedly higher in ELP-treated defects, while Collapat^®^ supported only sparse vascular ingrowth and showed no evidence of neuronal invasion (Figure 5). Neurofilament staining at early time points (weeks 1 and 2) reinforced these findings. In the ELP-treated group, axons were observed penetrating deep into the defect, whereas in the Collapat^®^ group, fibers remained confined to the periphery. In the empty group, the few neurofilament-positive fibers observed were primarily located at the edges of the defect and appeared to originate from adjacent muscle tissue, which tends to migrate into the defect site in the absence of a biomaterial. Thus, these fibers likely represent peripheral tissue migration rather than genuine innervation of regenerating bone.

3D tissue clearing and immunolabeling further supported these results. Endomucin^+^, Podocalyxin^+^, and MECA32^+^ vessels were distributed throughout the ELP-treated volume, while Collapat^®^ sites showed reduced infiltration. Additionally, 3D imaging confirmed peripheral and intradefect recruitment of nerve fibers exclusively in the ELP group (Supplementary Videos S1–S6). In empty defects, blood vessels were present but did not progress significantly over time (Figure 3C), and nerve fibers remained peripheral (Figures 4 and 5).

The structural differences observed between empty and ELP-treated defects highlight the role of the scaffold in directing not only bone formation but also tissue organization. In the absence of biomaterial support, bone regeneration appeared disorganized and poorly vascularized, whereas the ELP matrix favored the formation of well-structured, lamellar bone enriched with osteocytes and functional vasculature. Moreover, although Collapat^®^ induces bone formation, the newly formed bone tissue does not have as organized lamellar bone structure as observed in the case of ELP-based matrices (Supplementary Figure S3). Such organization is suggestive of more mature and biomechanically competent bone tissue. Importantly, the co-occurrence of vascular, neural, and osteogenic structures within the same spatial domains strongly supports the concept of neuro-osteogenic coupling [49], a phenomenon particularly relevant in sensory-rich craniofacial regions where coordinated regeneration of multiple tissue types is essential.

In the minipig model, which closely mimics human craniofacial structure [31], ELP-treated defects showed well-distributed and organized neoformed bone tissue by week 8 extending from the periphery to the center of the lesion. Histology evidenced the presence of lamellar bone and osteocytes and showed good integration of the ELP matrix into the host tissue, while Xenograft-treated sites retained large amounts of scaffold and exhibited more peripheral, less structured osteoid deposition (Figure 6A and Supplementary Figure S4). Quantitative analysis at week 8 (Figure 6C) revealed comparable levels of neoformed bone between ELP (36.3%) and Xenograft (36.2%) groups, without statistically significant difference.

However, histological differences pointed to a more advanced state of tissue maturation in the ELP group, including better-organized lamellar bone, deeper scaffold infiltration, and more consistent remodeling activity. Notably, early signs of medullary tissue formation were observed in ELP-treated sites by week 8, including vascularized areas containing adipocyte-like cells. This aligns with preclinical studies in large animals, where reconstitution of marrow-like spaces typically begins between 8 and 12 weeks, following the establishment of trabecular bone and vascular networks [50]. These features may reflect a regenerative trajectory that more closely resembles physiological bone healing. This observation is consistent with previous studies [51, 52] highlighting that complete bone regeneration involves not only mineralized matrix deposition but also the restoration of marrow spaces capable of supporting hematopoiesis and metabolic bone functions. This histological evidence further illustrates how scaffold architecture and bioactivity influence not only the extent but also the quality of regeneration, a critical aspect when designing biomaterials for load-bearing craniofacial applications. Although the results are encouraging, some limitations should be noted. The animal models used, while informative, do not fully capture the complexity of human craniofacial defects. In addition, although vascular and neural structures were clearly observed in ELP-treated sites, their long-term functionality remains to be demonstrated.

Despite these considerations, the strong regenerative performance of the ELP matrix, at least comparable to commercially available materials used in clinical practice in terms of bone formation, but with clearly superior efficacy in terms of tissue vascularization and innervation, supports its relevance as a new innovative and clinically promising scaffold.

## Conclusion

Altogether, the ELP-based matrix represents a significant advancement in craniofacial bone regeneration, driven by three key innovations. Its modular architecture, functionalized with bioactive peptides, enables the coordinated promotion of osteogenesis, angiogenesis, and innervation, effectively replicating the complexity of the native bone microenvironment.

Across two complementary preclinical models, the ELP matrix achieved increases in bone mineralized tissue, supported by neurovascular infiltration without the addition of exogenous cells or growth factors, and showed no inflammation. Compared to standard clinical materials, it not only fosters more organized neurovascularized bone formation but also enables high-resolution monitoring of bone healing progression.

These findings confirm that this modular, bioactive scaffold is not only safe and effective but also ideally positioned for clinical translation.

## Supplementary Material

rbag036_Supplementary_Data
