# Supplementary material for: A neurovascularized bone regeneration strategy for mandibular and alveolar bone defects based on elastin-like biomaterials
Source: Regen Biomater. 2026 Mar 5;13:rbag036. doi: 10.1093/rb/rbag036 (PMC13038255; doi:10.1093/rb/rbag036)
Supplement: rbag036_Supplementary_Data [file rbag036_supplementary_data.zip › SUP_material.pdf]

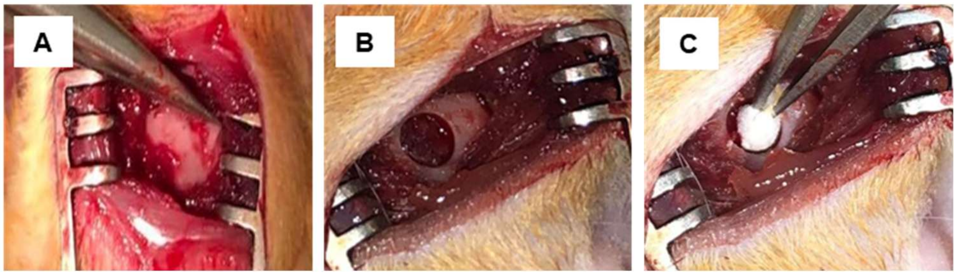

Figure Supplementary 1. Surgical procedure for mandibular bone defect creation in rats.

A. The surgery began with an incision to expose the rat mandible.
B. followed by the creation of a 3.3 mm defect using a trephine drill and removal of the bone segment.
C. The defect was then filled with the test biomaterial**.**

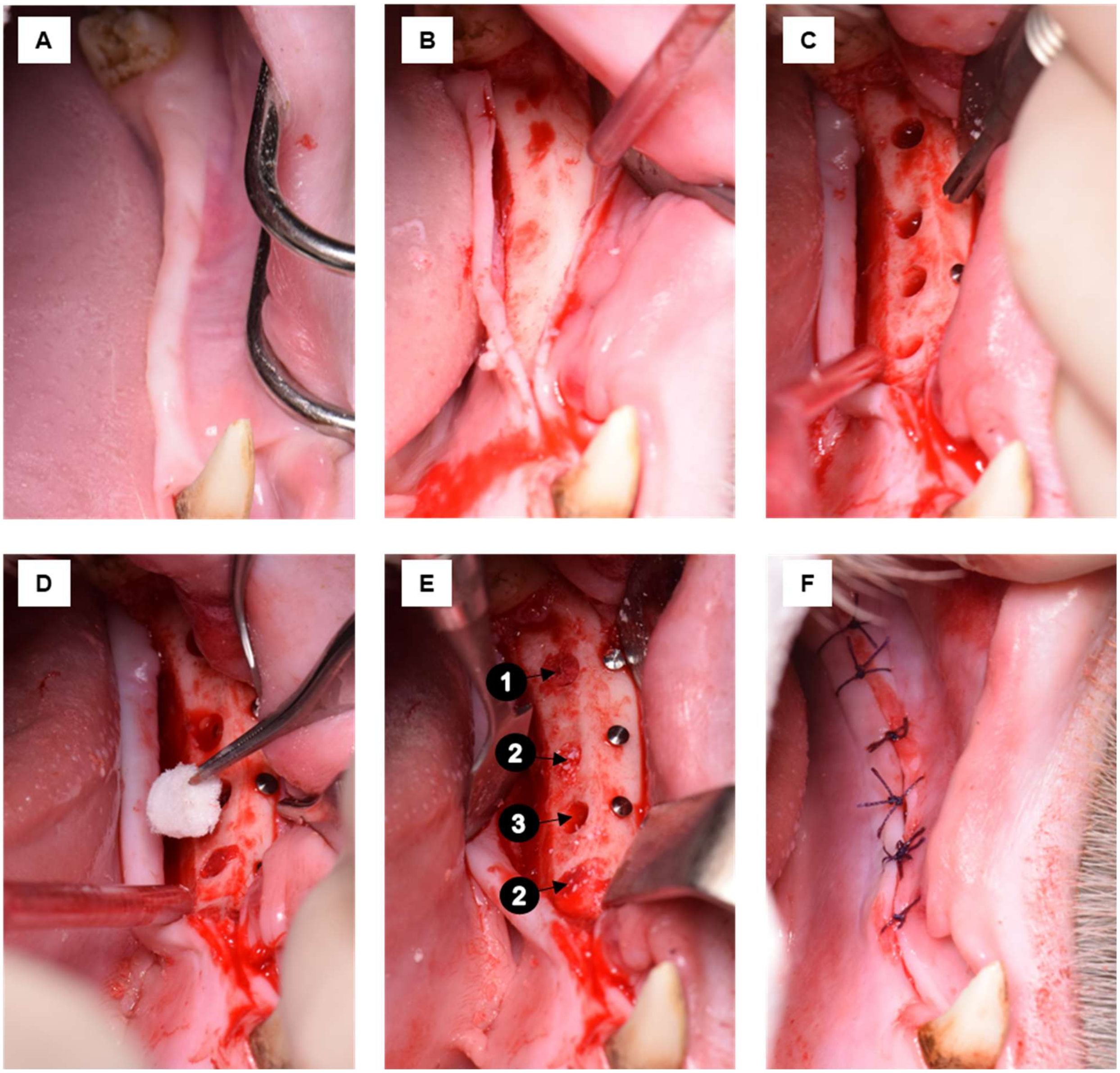

Figure Supplementary 2. Surgical procedure for alveolar bone defect creation in minipigs. Illustrations of the surgical procedure performed on a mini-pig for the creation and filling of an alveolar bone lesion.

A. Lateral view of the healed mandibular ridge prior to surgery.

B. The exposed mandibular implantation site after mucoperiosteal flap elevation and alveolar bone crest flattening.

C. Osteotomy was performed to create 4 implantations sites per hemi-mandible.

D. Implantation of the ELP matrix in one of the bone lesions.

E. From top to bottom, the lesions were filled with the ELP matrices (1), the second and fourth lesions filled with the xenograft (2), and the third left empty (3).

F. Mandible after suturing.

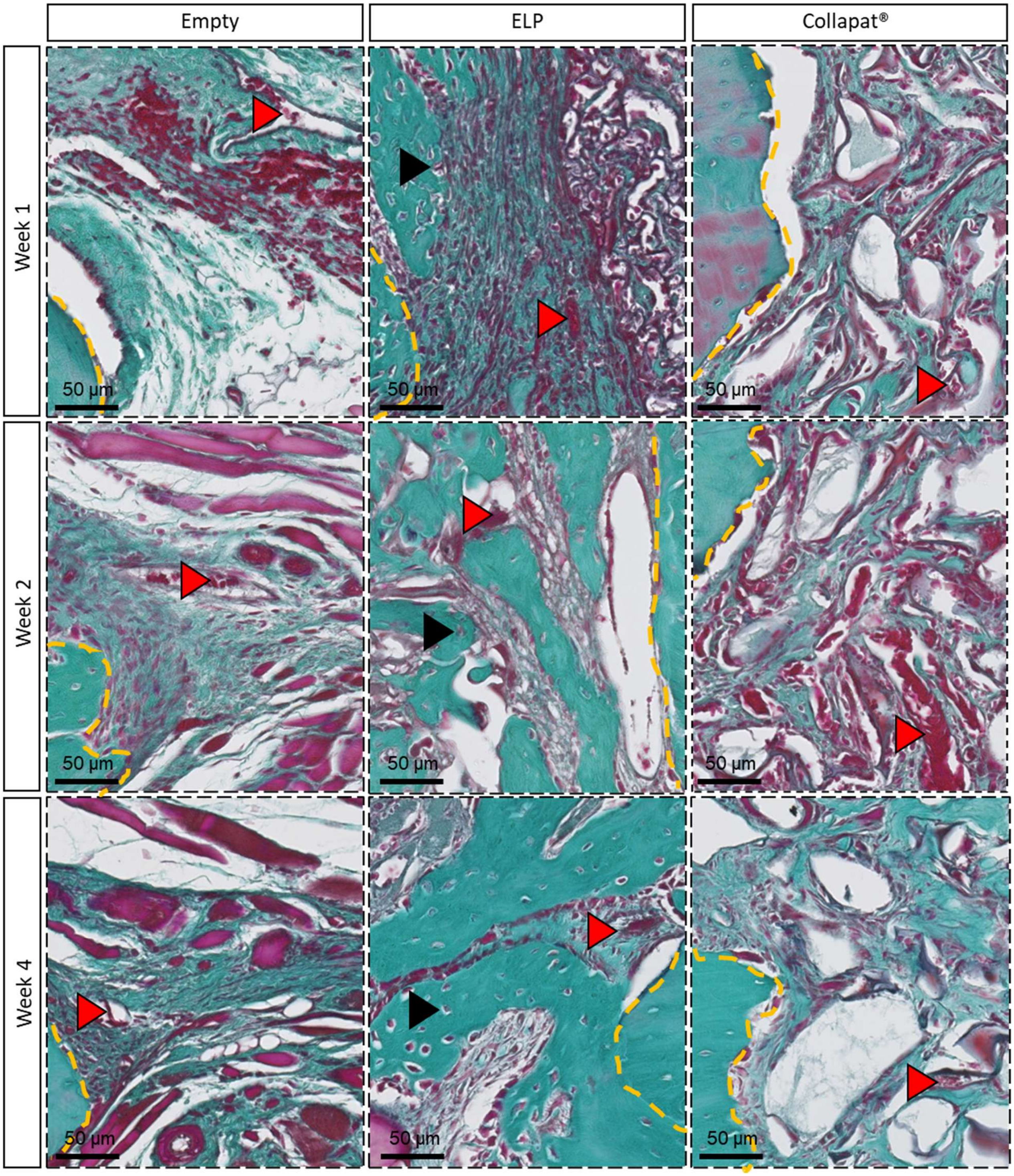

Figure Supplementary 3. Temporal histological assessment of bone regeneration in rat mandibular defects.

High-magnification views of representative Masson's trichrome-stained sections of mandibular bone defects at 1-, 2-, and 4-weeks post-implantation for the three groups: empty defect, ELP matrix, and Collapat®. The yellow lines delimit the area corresponding to the native bone tissue. Red arrow heads indicate blood vessels and black arrow heads indicate osteocytes in the newly formed tissue. Scale bars: 50 µm.

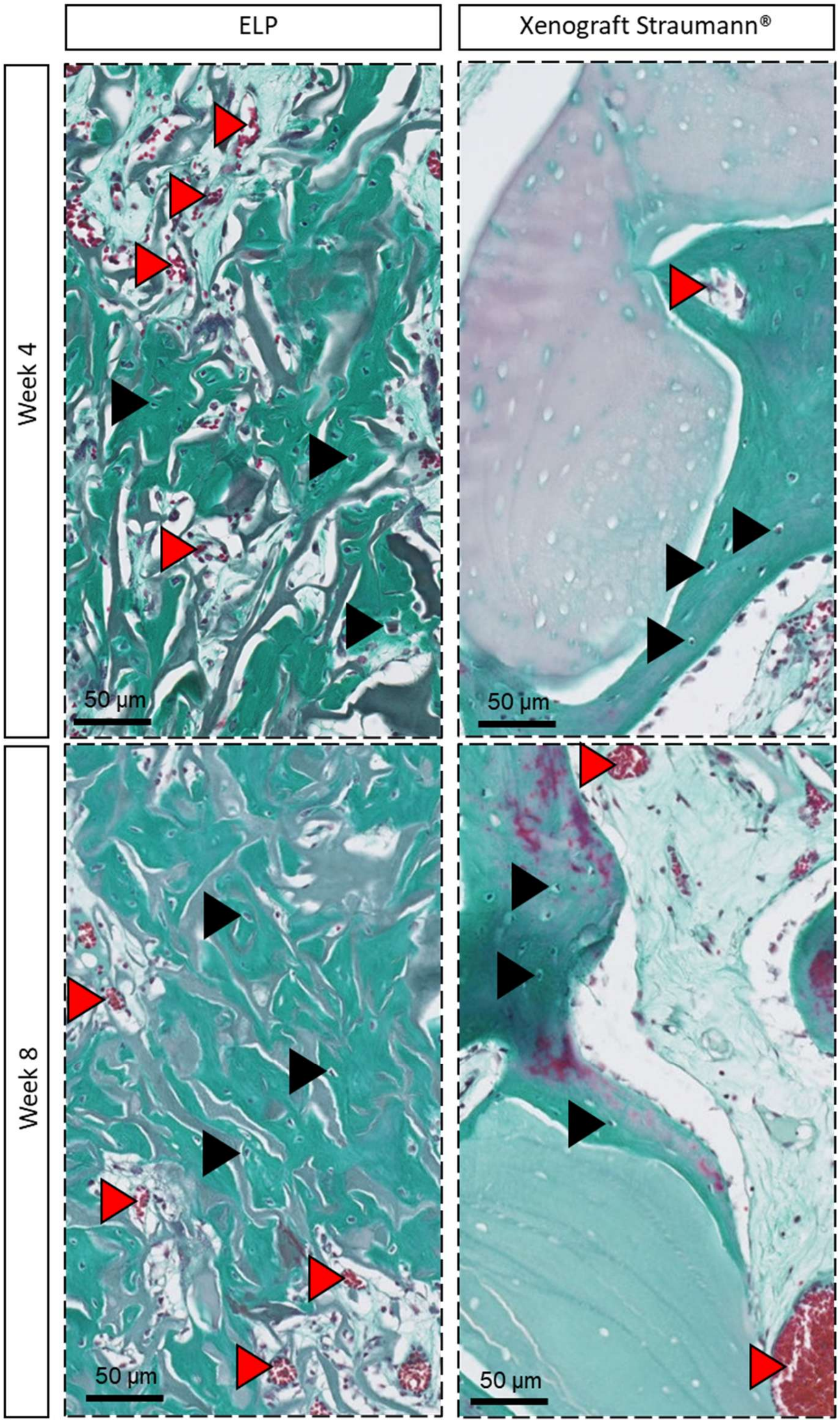

Figure Supplementary 4. Histological evaluation of alveolar bone healing in the mini-pig model.

High-magnification views of representative Masson's trichrome-stained sections of alveolar bone defects at 4- and 8-weeks post-implantation with the ELP composite matrix and the Xenograft Straumann® granules, shown in Figure 6. Red arrow heads indicate blood vessels and black arrow heads indicate osteocytes in the newly formed tissue. Scale bars: 50 µm.

Video Supplementary 1. 3D reconstruction of the entire empty mandible lesion, imaged by light-sheet microscopy after 2 weeks of implantation in the rat mandible lesion model. β3-Tubulin staining is shown in red to evidence the neural network. In cyan the staining of Endomucin, Podocalyxin and MECA32 to reveal the vascular network.

Video Supplementary 2. 3D reconstruction of the entire mandible lesion filled with the ELP matrix, imaged by light-sheet microscopy after 2 weeks of implantation in the rat mandible lesion model. β3-Tubulin staining is shown in red to evidence the neural network. In cyan the staining of Endomucin, Podocalyxin and MECA32 to reveal the vascular network.

Video Supplementary 3. 3D reconstruction of the entire mandible lesion with Collapat®, imaged by light-sheet microscopy after 2 weeks of implantation in the rat mandible lesion model. β3-Tubulin staining is shown in red to evidence the neural network. In cyan the staining of Endomucin, Podocalyxin and MECA32 to reveal the vascular network.

Video Supplementary 4. 2D flythrough of the entire empty mandible lesion, imaged by light-sheet microscopy after 2 weeks of implantation in the rat mandible lesion model. β3-Tubulin staining is shown in red to evidence the neural network. In cyan the staining of Endomucin, Podocalyxin and MECA32 to reveal the vascular network.

Video Supplementary 5. 2D flythrough of the entire mandible lesion filled with the ELP matrix, imaged by light-sheet microscopy after 2 weeks of implantation in the rat mandible lesion model. β3-Tubulin staining is shown in red to evidence the neural network. In cyan the staining of Endomucin, Podocalyxin and MECA32 to reveal the vascular network.

Video Supplementary 6. 2D flythrough of the entire mandible lesion with Collapat®, imaged by light-sheet microscopy after 2 weeks of implantation in the rat mandible lesion model. β3-Tubulin staining is shown in red to evidence the neural network. In cyan the staining of Endomucin, Podocalyxin and MECA32 to reveal the vascular network.
